# Comparative Genomics of Natural Killer Cell Receptor Gene Clusters

**DOI:** 10.1371/journal.pgen.0010027

**Published:** 2005-08-26

**Authors:** James Kelley, Lutz Walter, John Trowsdale

## Abstract

Many receptors on natural killer (NK) cells recognize major histocompatibility complex class I molecules in order to monitor unhealthy tissues, such as cells infected with viruses, and some tumors. Genes encoding families of NK receptors and related sequences are organized into two main clusters in humans: the natural killer complex on Chromosome 12p13.1, which encodes C-type lectin molecules, and the leukocyte receptor complex on Chromosome 19q13.4, which encodes immunoglobulin superfamily molecules. The composition of these gene clusters differs markedly between closely related species, providing evidence for rapid, lineage-specific expansions or contractions of sets of loci. The choice of NK receptor genes is polarized in the two species most studied, mouse and human. In mouse, the C-type lectin-related Ly49 gene family predominates. Conversely, the single Ly49 sequence is a pseudogene in humans, and the immunoglobulin superfamily KIR gene family is extensive. These different gene sets encode proteins that are comparable in function and genetic diversity, even though they have undergone species-specific expansions. Understanding the biological significance of this curious situation may be aided by studying which NK receptor genes are used in other vertebrates, especially in relation to species-specific differences in genes for major histocompatibility complex class I molecules.

## Introduction

Natural killer (NK) cells destroy cells infected with certain viruses and other intracellular pathogens. They also influence immune responses through the release of cytokines. A key aspect of recognition of appropriate target cells is the ubiquitously expressed major histocompatibility complex (MHC) class I molecule, a ligand for which NK cells generally have multiple receptors [1]. All living jawed vertebrates studied to date have an adaptive immune system, which, among other features, rearranges T and B cell receptor gene segments and exploits antigen presentation provided by MHC molecules [2,3]. Evolutionarily intermediate species, which lacked these features, presumably became extinct through competition with other species possessing a viable adaptive immune system [4]. A recognition-based MHC system may have been pivotal to survival of gnathostome species [3]. NK receptor gene complexes are intimately associated, both genetically and functionally, with MHC recognition, and interactions of different combinations of NK receptors and MHC class I molecules may contribute significantly to selection and disease resistance [4,5].

**Figure 1:**
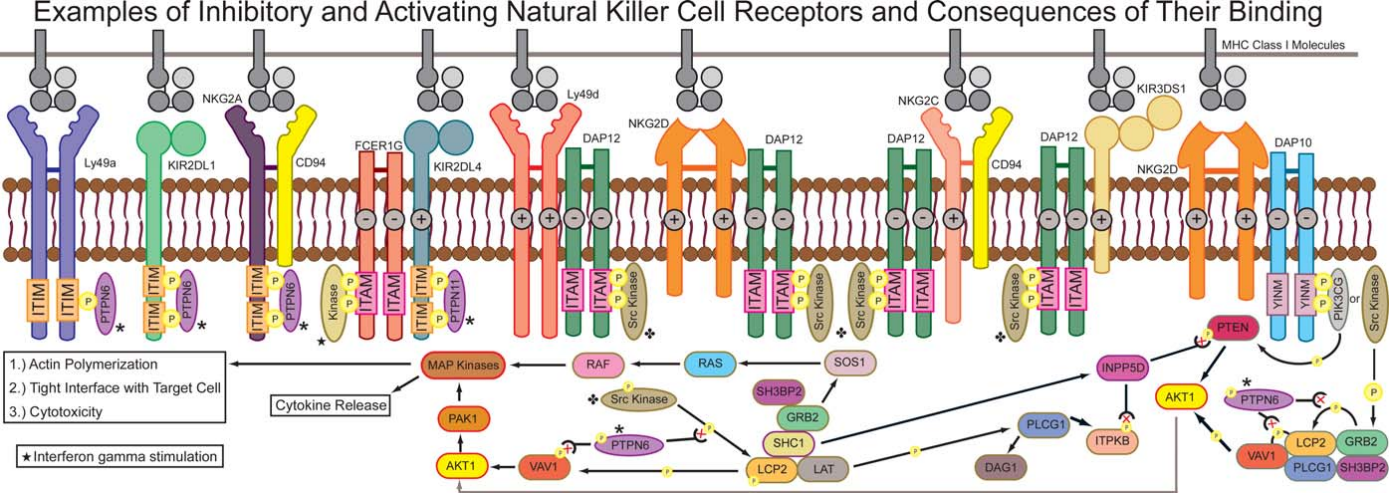
Examples of Inhibitory and Activating NK Cell Receptors and the Consequences of Their Binding This figure shows the association of NK receptors with MHC class I molecules and portions of the resulting signaling pathway, as described in the text. “Src Kinase” represents various kinases capable of participating in this interaction, such as Syk and ZAP70. SHP2 (PTPN11) is used in place of SHP1 (PTPN6) in some circumstances, such as associating with KIR2DL4. Symbols show the continuation of the pathway where arrows are not drawn. −, negatively charged residue; +, positively charged residue; AKT1, v-akt murine thymoma viral oncogene homolog 1 (also called RAC); DAG1, dystroglycan 1; FcɛRI-γ, receptor for Fc fragment of IgE, high affinity I, gamma polypeptide; GRB2, growth factor receptor-bound protein 2; INPP5D, inositol polyphosphate-5-phosphatase, 145kDa (also called SHIP); ITPKB, inositol 1,4,5-trisphosphate 3-kinase B (also called IP3KB); LAT, linker for activation of T cells; LCP2, lymphocyte cytosolic protein 2 (also called SLP76); P, phosphate group; PAK1, p21/Cdc42/Rac1-activated kinase 1 (STE20 homolog, yeast); PIK3CG, phosphoinositide-3-kinase, catalytic, gamma polypeptide (also called phosphatidylinositol 3-kinase); PLCG1, phospholipase C, gamma 1; PTEN, phosphatase and tensin homolog; PTPN6, protein tyrosine phosphatase, nonreceptor type 6 (also called SHP1); RAF, raf protein; RAS, rat sarcoma viral oncogene homolog; SH3BP2, SH3 domain–binding protein 2; SHC1, SHC (SH2 domain–containing) transforming protein 1; SOS1, son of sevenless homolog 1; VAV1, vav 1 oncogene; YINM, tyrosine-containing motif (YINM).

NK cell receptors come in two forms: inhibitory and activating. Inhibitory receptors regulate NK actions by interrupting intracellular activation signals when MHC class I molecules are correctly expressed [6]. Activating receptors, some of which bind ligands other than MHC class I molecules, trigger NK responses to cells with viral, bacterial, or parasitic infections or to some tumor cells with downregulated MHC class I molecules [7]. The effector function of each receptor molecule is determined by the sequence of its transmembrane region and cytoplasmic tail [8]. Generally, inhibitory receptors possess an immunoreceptor tyrosine-based inhibitory motif (ITIM) in their cytoplasmic tails [6], which decreases activation [9]. Upon stimulation, the ITIM becomes tyrosine phosphorylated and associates with intracellular phosphatases such as Src homology 2 (SH2) domain–containing protein tyrosine phosphatase 1 (SHP1) or SHP2. SHP1 then dephosphorylates the actin cytoskeleton regulator, Vav, which blocks actin-dependent activation signals [9]. In contrast, activating receptors lack ITIMs in their cytoplasmic tails and often contain charged residues that facilitate association with adaptor molecules containing immunoreceptor tyrosine-based activation motifs (ITAMs) such as DAP12 [10,11]. When activating receptors are associated with ITAM-containing adaptor molecules, the adaptor molecules become tyrosine phosphorylated and bind to kinases, which then interact with Vav and Rac1. The subsequent molecular cascade leads to actin polymerization and, consequently, cytotoxicity and/or cytokine release [11]. The ITIM/ITAM paradigm of inhibition/activation is a feature shared by NK receptors in all species, but it does not apply to all receptors. The human KIR2DL4 and KIR3DL3 molecules, for example, have unconventional cytoplasmic tails, and their mode of action, inhibition or activation, has not been clearly established [12]. Examples of inhibitory, activating, and co-stimulatory NK receptors, as well as the proposed pathways they initiate after ligand binding, are illustrated in Figure 1.

Genes that encode NK receptors are arranged in two main clusters: the leukocyte receptor complex (LRC) and the natural killer complex (NKC). The LRC encodes members of the immunoglobulin superfamily (IgSF), while the NKC encodes type II transmembrane, C-type lectin-like proteins [13]. The extent that receptors from each complex are expressed and utilized varies markedly among species. Comparing the genomic composition of NK receptor gene clusters in different species may provide clues to their evolution. The goal from these studies is to understand the selective forces, particularly in relation to disease, that have driven such extreme genomic differences. The diverse arrangements of genes encoding the MHC ligands of NK receptors [3] have to be taken into consideration, as NK receptors and their ligands coevolve.

## Human NK Receptor Gene Complexes

**Figure 2:**
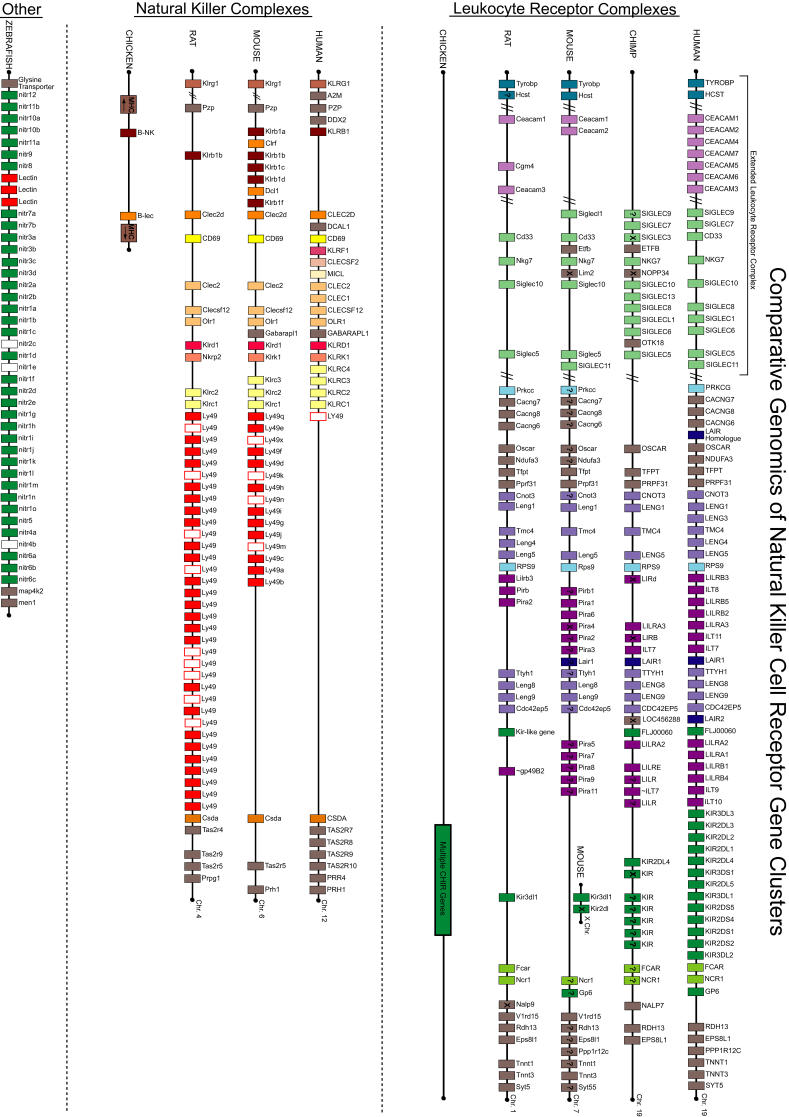
Comparative Genomics of Natural Killer Cell Receptor Complexes This figure, showing LRCs and NKCs in various species, is not drawn to scale; however, the linear arrangement of genes is correct and is aligned vertically by homology within each region, where possible. Colors indicate genes related by gene organization, structure, and phylogeny. Gray indicates genes that are not considered NK receptors. White boxes mark pseudogenes. Slash marks represent large distances in the genomic sequence. Some non-NK receptor genes that are located within this region may not be represented in this figure. A question mark indicates that the gene has been mapped to the corresponding chromosome, but the specific chromosomal position is not known. An “X” indicates that the gene is not homologous with genes sharing its vertical alignment. The linear organization of these genes was taken from literature sources as described in the text and from NCBI's MapViewer [123]. Similar to in human, only one Ly49 gene has been found in baboon, orangutan, dog, cat, cow, and pig. However, the horse has multiple Ly49 genes, as does the rodent lineage [96]. The chicken contains two sequences similar to NKC-encoded genes within its MHC [102], again suggesting an evolutionary relationship between MHC and NK receptor genes. The chicken also possesses multiple genes for Ig domain–containing NK receptors: the CHIR genes [100]; however, the chicken arrangement is from data assembled from whole genome sequence and will be refined as more accurate annotation is applied. While the zebrafish contains NK receptors containing Ig or C-type lectin domains, the genomic organization does not resemble the mammalian pattern of clustering into two main regions [104].

The main NK receptors for MHC class I molecules in humans belong to the IgSF and are encoded in the LRC on Chromosome 19q13.4 [14]. Of the 45 genes in the LRC, the 30 IgSF receptors can be grouped into several related gene families based on gene organization, phylogeny, and structure [15]. These families include the killer cell immunoglobulin-like receptors (KIRs), leukocyte Ig-like receptors (LILRs; also called LIRs and ILTs), and the leukocyte-associated Ig-like receptors (LAIRs). The most centromeric end of the human LRC contains genes for the platelet glycoprotein VI (GP6), the natural cytotoxicity-triggering receptor 1 (NCR1; also called NKp46), and the receptor of the IgA Fc fragment (FCAR; also called CD89). These proteins are structurally similar to KIR genes but differ from other loci in the LRC by interacting with ligands other than MHC class I molecules [15]. The organization of the human LRC is shown in the comparative maps of Figure 2.

The key LRC genes directing NK cell recognition of MHC class I are the KIRs. The KIR gene cluster exhibits marked differences in gene content and allelic polymorphism between individual haplotypes [16,17], with up to 17 KIR genes and pseudogenes arranged tandemly over approximately 150 kilobases [4]. KIR genes share high sequence similarity (most are approximately 97% identical), exhibit marked levels of polymorphism, and evolve rapidly [18]. This last characteristic may be facilitated by nonreciprocal crossovers in the tandemly arranged genes, occasionally generating hybrid loci or expansion/contraction of gene numbers [18,19]. The KIR gene family in the human/primate lineage appears to have expanded between 31 and 44 million years ago [13], as dated by the amplification of retroelements of the Alu S subfamily found in the KIR genomic sequence [17]. KIRs can possess either two (KIR2D) or three (KIR3D) extracellular Ig domains [15] and comprise long forms, which contain inhibitory ITIMs, or short forms, which lack ITIMs and are mostly activating [18]. KIR2DL4, however, is unique, having both putative inhibitory and activating properties [12]. In addition to possessing ITIMs in its cytoplasmic tail, KIR2DL4 has a charged arginine residue in the transmembrane region that allows association with the activating accessory protein FcɛRI-γ (FCER1G) [20].

Centromeric to the KIR family is another extensive IgSF gene family, the LILRs. This family consists of two inverted, duplicated clusters of six and seven loci, respectively [21]. The LILR genes show greater intergenic diversity, but less allelic variation than the KIR family [15]. Unlike KIR genes, there is little variation in the number of LILR genes on different haplotypes. Separating the LILR clusters are two LAIR loci, which sandwich members of the LRC-encoded novel gene (LENG) family. It has been proposed that the LAIR locus was duplicated and inverted with the LILR expansion, but the LRC-encoded novel genes are structurally unrelated to other Ig-like receptors in the LRC and have a different origin [15].

Additional genes related to those in the LRC are dispersed further centromeric of the LILR and KIR gene families in the “extended LRC” region. This region includes the receptor and transporter of the Fc fragment of IgG (FCGRT), which interestingly may have a common origin with MHC class I molecules [22], the sialic-acid-binding immunoglobulin-like lectin (SIGLEC) gene family [23], and the CD66-related gene family [24]—all of which are related to LILR and KIR sequences [13].

The other major cluster of NK receptor genes, the human NKC, is located on Chromosome 12p13.1 [25] and encodes mainly disulphide-linked dimeric, type II transmembrane molecules with homology to C-type lectins [26]. NKC-encoded genes have highly related genomic structures and are organized into distinct clusters of related genes [26]. Comparative maps of NKCs are shown in Figure 2.

The NKC contains a variety of C-type lectin genes, some of which are expressed specifically on NK cells. There is only one member of the Ly49 gene family, *KLRA1* (also called *Ly49L*), in humans, as opposed to the multiple homologous genes encoding MHC class I ligands in rodents [27]. While *KLRA1* is transcribed, a point mutation causes the production of a nonfunctional molecule [27]. Another gene family expanded in rodents but with only one human homolog is *KLRB1A* (also called *NKRP1A*). NKRP1A is expressed only on a subset of NK cells and T cells, as opposed to all NK cells in rodents [28].

Members of the NKG2 *(KLRC)* family of molecules dimerize with the linked, invariant CD94 molecule *(KLRD1)* on the cell surface, which as a partner chain provides the appropriate signaling motifs [29]. Some *KLRC-*encoded molecules, namely NKG2A *(KLRC1)* and NKG2C *(KLRC2),* signal as a result of binding to the nonclassical MHC class I molecule HLA-E [30]. This family encodes both inhibitory (NKG2A, NKG2B, and KLRL1) and activating (NKG2C, NKG2E *[KLRC3],* and NKG2H) receptors, some of which, such as NKG2B, NKG2E, and NKG2H, are products of alternative splicing [26]. When both inhibitory and activating NKG2 receptors are coexpressed on the cell surface, the inhibitory molecules appear to be functionally dominant, which may relate to the fact that they have a higher-affinity binding [31]. The NKG2 family also contains a unique receptor, NKG2F *(KLRC4),* that has a charged residue in the transmembrane region, an ITIM-like domain, and no C-type lectin–like domain [32]. The function of this molecule is not known.

NKG2D *(KLRK1)* shows limited sequence identity to other NKG2 molecules and is expressed on NK cells, T cells, and macrophages as a homodimer [26]. For activation, NKG2D signals by associating with DAP10, a molecule whose Src homology 2 domain recruits the p85 subunit of phosphatidylinositol 3-kinase (PI3K) [33,34]. Human NKG2D binds MHC class I chain-related protein (MIC) A, MICB, and the UL-16 binding protein (ULBP) family [35].

KLRF1, which is present in humans but not mice [26], stimulates NK cells upon cross-linking. The inhibitory-receptor-encoding *KLRG1,* also called mast cell function–associated antigen [36], is more centrally located in the human NKC than in that of the mouse and may have arisen by gene duplication and chromosomal inversion events [26]. Other NK surface molecules encoded in the human NKC include the activation-induced C-type lectin (AICL) [37], lectin-like transcript 1 (CLEC2D; also called OCIL and LLT1) [38], and CD69 [26]. Genes that encode products not found on NK cells, which are also located within the NKC, include alpha-2-macroglobulin (A2M) [26], oxidized low-density lipoprotein (lectin-like) receptor 1 (OLR1) [39], CLECSF12, CLEC1, and CLEC2 [40].

## Nonhuman Primate NK Receptor Gene Complexes

KIR genes have diverged dramatically between different primate species, consistent with rapid, species-specific expansion of the gene family [41]. In chimpanzees, the seven KIR genes, of which only three *(KIR2DL4*, *KIR2DL5*, and *KIR2DS4)* are best reciprocal human orthologs, cover 106 kilobases [41,42]. In the gorilla, 11 KIR genes have been identified with two genes being orthologous to human KIRs [43]. Orangutans, which diverged earlier from humans in the primate lineage than chimpanzees and gorillas, also have a species-specific expanded KIR repertoire [44]. The rhesus macaque only has five KIR genes [45,46]. The African green monkey *Chlorocebus sabaeus,* another Old World primate more closely related to rhesus monkeys than to the apes, possesses multiple KIR genes, namely *KIR3DL, KIR2DL4, KIR2DL5,* and *KIR3DH,* a KIR form found in rhesus and African green monkeys but not apes, and a hybrid of *KIR2DL5* and *KIR3DH* [47]. *KIR2DL4* is the only orthologous KIR gene found in humans, chimpanzees, gorillas, rhesus macaques, and African green monkeys. Interestingly, not all gorillas appear to have *KIR2DL4* [43], and it is nonfunctional as a receptor in orangutans [44]. Table 1 shows, where known, the differences in gene content of NK receptor genes among various species.

**Table 1:**
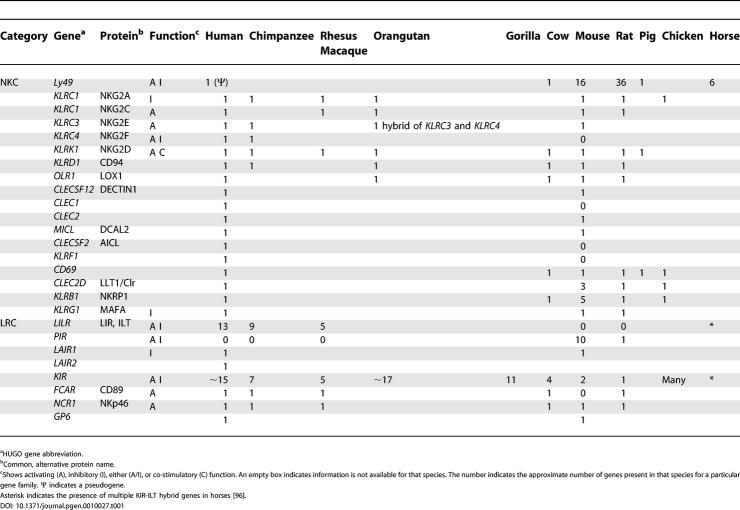
Presence of NKC and LRC Encoded Genes in Different Species

^a^HUGO gene abbreviation.

^b^Common, alternative protein name.

^c^Shows activating (A), inhibitory (I), either (A/I), or co-stimulatory (C) function. An empty box indicates information is not available for that species. The number indicates the approximate number of genes present in that species for a particular gene family. Ψ indicates a pseudogene.

Asterisk indicates the presence of multiple KIR-ILT hybrid genes in horses [96].

These differences in KIR gene presence accord with differences in MHC class I gene content among primate species [48]. For example, HLA-C and its equivalent in other primate species differentiated from an HLA-B-like ancestor after divergence of the hominoid and monkey lineages [4,49]; therefore, it is only present in primate species more closely related to humans. While HLA-C is consistently present in humans and chimpanzees (the closest relative of humans), it is absent in approximately half of the orangutan haplotypes, and when it does occur in orangutans, it resembles an evolutionary intermediate of human HLA-C [4,44]. Furthermore, Popy-C, the orangutan HLA-C equivalent, provides only one of the two MHC-C motifs that are used to control human and chimpanzee NK cells [42,50]. Dimorphism at amino acid 80 defines these two groups, where MHC-C1 has Asn80 and MHC-C2 has Lys80. The receptors for the C1 and C2 groups are the inhibitory KIR2DL molecules: KIR2DL2 and KIR2DL3 interact with C1, while KIR2DL1 interacts with C2 at a higher affinity. From these primate evolutionary studies, we have learned that the weaker C1–KIR2DL2/3 interaction arose first, and the stronger C2–KIR2DL1 interaction arose later. Since both C1 and C2 allotypes are represented in all human populations, these molecules may have complementary functions that enable the strength of HLA-C-mediated inhibition to be varied. This situation has implications for MHC/KIR combinations in disease, as has been evidenced by several recent papers [51–55]. The association of combinations of C1/C2 types and KIR alleles with predisposition to preeclampsia suggests other selection mechanisms [56]. The reciprocal levels of HLA-C1/2 allotypes and their respective KIR ligands in different human populations is consistent with a balance of advantages and disadvantages in level of response, presumably depending on local parasites and environmental conditions.

LILR genes are present in all primates studied, and although the gene content of this family shows greater differences between primate species than is observed throughout the genome, LILR genes are less variable in gene number between haplotypes than the KIRs. In chimpanzee, the nine LILR genes border the KIR sequences and are arranged in two duplicated clusters similar to human [41,57]. Four of these genes are orthologs of human LILR sequences [57]. The rhesus macaque has five LILR sequences [41]. Other NKC genes known to be present in the chimpanzee and rhesus macaque are orthologs of *FCAR* and *NCR1* [41].

There is limited information on NKC genes in primates. Members of the NKG2 family and *CD94* have been found in chimpanzees [42], including orthologs for all members except *KLRC2,* for which there are two paralogs [58]. Rhesus monkeys possess *NKG2A, NKG2B, NKG2C, NKG2D,* and several splice variants [45]. In the orangutan, orthologs for human *CD94, NKG2A, NKG2D,* and *NKG2F* are present, along with a hybrid gene combining *NKG2C* and *NKG2E* midway between their expected syntenic positions [44]. Orangutans also possess a functional homolog of *KLRA1* (Ly49L) [44], which is a pseudogene in humans [27,59]. A single *Ly49* sequence, which appears to be functional, has also been reported in baboons [60].

## Rodent NK Receptor Gene Complexes

The mouse LRC is located on Chromosome 7 [15], although it contains none of the KIR loci that form a cornerstone of MHC class I recognition in humans. However, two murine KIR-like sequences have been detected outside the LRC on the X chromosome [61,62]. There are conflicting reports on the function of one of these murine KIR-like sequences: one study found that Kir3dl1 (Kirl1) lacks an ITIM and any residue capable of binding to an activating adaptor molecule [61], while another reported the presence of two ITIMs in Kir3dl1 [62]. Surprisingly, the other KIR-like sequence, Kirl2, is selectively expressed in defined areas of the mouse brain [63]. The murine LRC contains orthologs of human *GP6* [13], *NCR1, RPS9,* and *LAIR1* [15]. The Pir gene family members (including *Pira1* to *Pira11* and *Pirb*), which share sequence identity with the human LILRs, are found between *Ncr1* and *Rps9,* [64]. In addition to sequence similarity, the Pir genes are arranged into two clusters similar to the LILRs and encode products that may interact with certain MHC class I molecules [65]. Genes of the murine LRC broadly display syntenic homology with the human arrangement apart from the absence of KIR loci.

In the rat, the LRC is located on Chromosome 1 and includes an ortholog of murine *Pirb, Ncr1* [15], and one KIR sequence (Kir3dl1) with a potential ITIM [62]. The rat also has *Fcar,* which, while also present in humans, was lost in the mouse lineage [66].

Orthologs of human NKC genes are reported in a syntenic region of murine Chromosome 6. The gene families are arranged in a similar order to the human NKC, except for expansions and interchanges of the C-type lectin related (Clr) (ortholog of human *OCIL*) and Nkrp1 (Klrb1) gene families [26]. Interestingly, the products of these two genetically intertwined families in mouse interact, suggesting potential functional advantages for their adjacent gene locations [67]. There are several Klrb1 (Nkrp1) genes in mouse, with Klrb1a, Klrb1c, and Klrb1f taking activating forms and Klrb1b and Klrb1d being inhibitory [26]. Members of the NKC-encoded C-type lectin related gene family have been found as ligands for Klrb1d and Klrb1f [67].

In the rat, the NKC is located on Chromosome 4. In fact, the first NKC gene discovered in any species was rat *Nkrp1* [68,69]. Human, mouse, and rat all share orthologs of *CD69* and *KLRD1* (CD94); however, there are still many differences between human and rodent in the gene content of the NKC [70] (see Figure 2; Table 1).

**Figure 3:**
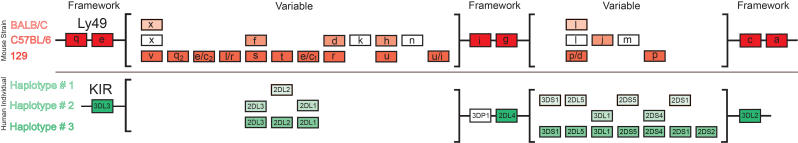
Differences in Organization of KIR Genes in Human Haplotypes and Ly49 Genes in Various Mouse Strains This figure is not drawn to scale, but it illustrates the presence of framework genes and the variable gene content in Ly49 genes in different mouse strains [72] and KIR sequences in human haplotypes. The vertical arrangement of genes within a family shows allelic relationships. White boxes indicate known pseudogenes. Please note that while many KIR haplotypes are possible, only a small selection has been shown here.

In contrast to the single human Ly49 locus, the polymorphic Ly49 gene family in mice contains at least 16 genes and pseudogenes [71], named *Ly49a* to *Ly49q,* although the gene content can differ significantly in different mouse strains [26]. While framework Ly49 genes are present in all murine haplotypes, the Ly49 gene content between these framework genes varies at some loci [72], in a similar manner to what is observed in the human KIR family. These framework/strain-specific differences are shown in Figure 3.

The Ly49 molecules have a variety of functions and expression patterns. Ly49a, Ly49c, Ly49g, and Ly49i contain ITIMs and act as NK inhibitory receptors when recognizing MHC class I molecules. Conversely, Ly49d and Ly49h exhibit activating properties. Ly49e functions in fetal NK cell development, when other Ly49 molecules are not present [26]. Ly49q is found on plasmacytoid dendritic cells and not NK cells [73,74].

In the rat, this family has expanded even further. The most recent report lists 19 functional rat Ly49 genes and 15 pseudogenes [75]. The rapid expansion of rat Ly49 genes appears to result from repeated tandem and block gene duplications, which occurred after species divergence from the mouse [76], and might be related to the higher number of MHC class I genes in the rat genome than in the mouse genome [77].

The Ly49 family in mouse parallels the human KIR both functionally and genetically [78]. Insight into the evolutionary drive behind gain or loss of Ly49 loci was provided by studying the relationship between Ly49 genes and murine cytomegalovirus (mCMV) [79–82]. When the Ly49h receptor in mice is not present, is blocked by monoclonal antibody, or has its pathway interrupted through mutation of the DAP12 molecule, uncontrolled viral replication of mCMV occurs [80,83], demonstrating that the function of this activating receptor is essential for mCMV resistance [26]. Ly49h interacts specifically with mCMV protein m157 to counteract inhibition imposed by the viral protein engaging Ly49i [84]—an example of the biological arms race [85].

The KLR gene family in mouse contains several members, including *Klrc1, Klrc2, Klrc3, Klrd1, Klri1,* and *Klri2* [26,86]. Similar to recognition of HLA-E in humans [30], members of this family recognize the mouse HLA-E functional homolog H2-T23 [86]. Nkg2d binds minor histocompatibility molecule H60, the retinoic acid early transcript 1 (Rae-1) family, and mouse UL-16 binding protein–like transcript 1 (Mult1). Interestingly, the Mill gene family, the murine functional equivalent of human MIC genes, which are encoded in the human MHC class I region and bind NKG2D, are found near the murine LRC [87]. In mice, Nkg2d is proposed to have activating properties through association with Dap12 and Dap10 [34,88]. Human NKG2D cannot associate with DAP12 to produce an activating signal [89]. A direct ortholog of KLRF1 is not present in the mouse. The murine NKC also contains *Klrg1* and *Cd69,* and genes other than those encoding NK receptors—genes such as *alpha-2-macroglobulin, Clecsf12,* and *Clec2* [26]. Genes encoding three Klrc molecules, Nkg2d, CD94, Klrb1, and Klrh1 (an inhibitory receptor) have been reported in rat.

## NK Receptor Gene Complexes in Other Species

Since the genomic organization of human and mouse class I receptors is so polarized, in terms of KIR or Ly49 gene content, respectively, it is informative to study other species. The bovine LRC, located on Chromosome 18, includes activating and inhibitory KIR sequences and an ortholog of *NCR1* [90]. Sequence similarity comparisons between cows and primates indicate that the multigenic KIR families expanded independently in the two lineages [91]. Known genes in the bovine NKC include *KLRK1, NKRP1* [92], *KLRJ1* [91], and one Ly49 gene [93]. There is a *CD69* transcript in cows as well, but its genetic location is not known [94].

Like humans, the single Ly49 sequence is recapitulated in cattle [93], domesticated cats, dogs, and pigs [95]. Like rodents [76], horses show multiple Ly49 genes: five that encode ITIMs and one with potentially activating properties [96]. The horse also has an expansion of a family of KIR-ILT hybrid genes [96]. These species with both multiple Ly49 and multiple KIR sequences indicate that the functions of the two sets of MHC class I ligands can be coordinated. The polarized arrangement in humans (all KIR) and mice (all Ly49) suggest that either (1) the existence of both sets within a single individual poses logistical problems, which have been solved by disabling one set, or (2) the human and mouse arrangements are outriders and in most other vertebrates the functions of both sets of genes synchronize well with each other. Clearly, the MHC class I/NK ligand gene arrangements of many other species need to be evaluated.

A gene complex encoding NK receptors in pig is located on Chromosome 5, with reports of direct orthologs for human *CD69* and *KLRK1* [97]. Chicken is a key species because of its “minimal essential” MHC [98]. A family of chicken Ig-like receptors (CHIRs) are related to Pirs and Fc receptors and are arranged in similar genomic clusters to human KIR and LILR genes [99]. Recent work has shown the presence of numerous CHIR genes in the chicken genome, with many receptors possessing both activating and inhibitory properties [100]. The multiple CHIR genes are thought to have emerged from a common ancestor with humans before the mammalian radiation and then expanded in a lineage-specific manner [100,101]. There are examples of chicken NK receptors, such as B-NK and N-lec, which contain C-type lectin domains, similar to the NKC-encoded NKRP1 and LLT1 in humans. It is interesting, though, that the genes encoding these proteins are located within the chicken MHC region, consistent with an ancient genetic relationship of these MHC and NK receptors [102].

The zebrafish, a teleost model organism, contains a cluster of putative activating and inhibitory NK receptors called the novel immune-type receptors (NITRs). While these genes encode an Ig domain, there is a cluster of C-type lectins encoded within this larger cluster [103,104]. Another bony fish, *Oreochromis niloticus,* possesses a KLR region, containing 26 genes, though more compact than its human counterpart [105]. Other teleosts have NKC genes, including *Paralabidochromis chilotes* [106] and rainbow trout [107], demonstrating that some of these genes arose in an ancestor common to both humans and bony fish earlier in the gnathostome lineage.

## Conclusions

As explained above, features of NK receptor genes both within and between species are consistent with rapid evolutionary change. Inevitably, studies focus on human KIR and mouse Ly49 genes, but there are indications from the few studies of other vertebrates that variable C-type lectin and IgSF receptors for MHC class I molecules may co-exist in a species, such as horse [96], or may be functionally replaced by another divergent family of genes, as may be the case in chicken [100,101]. The marked expansion of the Ly49 gene family [76] and the large differences in gene number and content between closely related species, such as the mouse and rat [108], attest to rapid evolution of NK receptor genes. The existence of only one Ly49 pseudogene in humans [27] while a homolog remains functional in other primates [44,60] also indicates rapid evolutionary change since the divergence of a common ancestor. It is possible that the presence of gene families such as those found in the NK receptor complexes facilitates rapid evolution through recombination, subfunctionalization of duplicated genes, and conservation of essential sequence [109].

Emerging data are also consistent with rapid evolution of NKC-encoded genes. These include comparisons of KIR haplotypes in chimpanzee, rhesus macaque, and human, where, in addition to gene content differences, repeat elements in intronic regions suggest rapid evolution [41]. Another recent report showed that the KIR and Ly49 gene families have among the highest expansion rates in the genome, with the human KIRs expanding by 0.52 genes per million years and rat Ly49 genes expanding by 0.54 genes per million years since duplicating from a single common ancestor [76]. A more conservative study to determine the genome-wide average for this rate found it to be 0.001 to 0.03 genes duplicated per million years [110]. Recent data indicate that inhibitory KIRs are ancestral and that their activating counterparts have evolved from them by mutation [111]. It appears that the development of activating versions of polymorphic receptors takes place in both KIR and Ly49 loci. Thus, in different species, convergent evolution results in activating genes with similar signaling domains. This mechanism is necessitated so that the receptors can couple with appropriate signaling adaptors (see Figure 1), which are ancient and much more conserved than both KIR and Ly49. The mechanism is streamlined, in that tails associated with activating adapters become associated with different receptors by nonhomologous recombination [19]. Interestingly, activating receptors appear to evolve recurrently, presumably in line with selection associated with resistance to disease.

Perhaps because of this “response mode” fluctuation in activating versions of inhibitory receptors, it has proved difficult to identify their role and indeed their ligands. An interesting exception is mouse Ly49h, which recognizes mCMV-infected cells by a direct interaction with the m157 mCMV gene product [84]. So far, no other activating receptors appear to be dedicated to pathogen-specific products. However, there is evidence for epistasis between MHC class I loci and Ly49 in resistance to mCMV, which may explain the difficulty in identifying ligands for activating receptors [112]. Ly49p is an activating receptor that specifically recognizes mCMV-infected cells but only in the context of H-2Dk. Accordingly, binding of Ly49p was blocked by antibodies to H-2Dk but not by those to H-2Kk. It is not known what lies behind the epistasis. It is possible that Ly49p recognizes H-2Dk only when certain viral peptides are present. It has been proposed that in the case of another receptor, Ly49c, certain peptides might exert interactions through the floor of the H-2Kb binding groove, which are transmitted to the NK receptor by β2 microglobulin [113]. Alternatively, NK receptors such as Ly49c and Ly49p might respond to an H-2 molecule in the presence of a host-encoded protein that is upregulated upon viral infection. Intriguingly, similar mechanisms could be put forward to explain the role of activating KIRs such as KIR2DS1 being upregulated during Epstein-Barr virus infection [114].

Why do some species expand KIR genes while others expand Ly49 genes? While these two gene families produce proteins of analogous function, they do not share a common ancestor. These expansions vary greatly even within lineages, such as between human and chimpanzee (diverged approximately 5 million years ago) or mouse and rat (diverged approximately 20 million years ago) [115]. It is tempting to assume that different life spans, environments, sizes, and, specifically, pathogen interactions influence fixation of different NK receptor repertoires in different species. NK receptors are important components of antiviral immune responses and are an essential bridge between early innate responses, such as the release of virally induced interferon-alpha (IFNA) and interferon-beta (IFNB), and the later T cell and antibody adaptive responses [116]. Viruses evolve rapidly to environmental conditions, crossing species boundaries and mutating quickly to allow success in specific host species [117]. It is possible that the rapid evolution and host-species-specific adaptations of viral pathogens, which are common targets of NK-mediated immune responses, could influence the rapid expansion of different NK receptor repertoire combinations among species. Selective forces other than infection may also be entertained, including autoimmunity and reproduction, illustrated by the link between preeclampsia and combinations of HLA-C in the fetus and KIR in the mother [56].

NK receptor gene clusters coevolve with MHC genes [3,15], and there are clues to genetic and functional relationships between them [1]. MHC class I–like molecules have directed the development of different lymphocytes throughout evolution, as demonstrated both by the presence of common cell surface markers on NK cells, γδ T cells, and CD8^+^ αβ T cells and by distinct receptors present exclusively on each class [4]. The large differences in genomic organization of NK receptor gene complexes between species, and between populations of humans, are likely driven by resistance to infection and exposure to different local pathogens [118,119], although different mechanisms are possible [120]. Some MHC-encoded genes maintain a large number of low-frequency alleles within the population [118], produce molecules that interact to control NK cell function, and evolve rapidly to maintain their epistatic interactions [42]. Ig domains provide an example of the way coevolution of these interactions may occur. The Ig domains encoded by LRC genes are IgC2 or vIg-like [121]. IgC2 domains appear to have evolved to recognize different Ig-like receptors [15], such as those found in the MHC, consistent with coevolution of some LRC and MHC genes. Another example of coevolution between NK receptor genes and MHC genes is provided by the Mill gene family in mice and rats. The Mill family members are functional homologs of human MIC genes, which are found in the human MHC class I region. However, in mice and rats, the Mill genes have translocated to a chromosomal area near the LRC [87], which, through linkage with NK receptor genes, could facilitate coevolution of polymorphisms affecting their epistatic interactions and enhance their transcriptional regulation. Furthermore, the coevolution of NK receptors and HLA-C molecules, observed in primates [44], could have implications for diseases [4,5] and for the interactions between HLA-C molecules and decidual NK receptors in the placenta [56].

Additional insights into the rapid coevolution of the NK receptor genes and genes encoding their MHC class I ligands can be gained by studying their levels of polymorphism. Publicly available polymorphism data, evaluated in a previous study from our laboratory [122], showed that MHC class I molecules possess extremely high levels of polymorphism, while the numbers of polymorphisms per kilobase for NK receptor genes are nearer to values for the rest of the genome. Although some NK receptor gene families have noticeably varied gene contents between haplotypes, NK receptor genes are not as highly polymorphic as MHC class I ligands. Therefore, we could assume that selective pressures, such as exposure to pathogen, drive the generation of genetic variation primarily on MHC class I genes. The variation in NK receptor gene content both within and between species, such as preference towards rapid expansion of KIR, Ly49, or both, could be a mechanism for coevolving with the rapidly evolving MHC class I molecules. This would explain how NK receptor gene complexes exhibit rapid evolution, measured by parameters such as gene gain and loss [76], while having moderate levels of sequence polymorphism. Given the essential interactions of MHC and NK receptor gene clusters, the high levels of polymorphism, and association of the MHC with disease, studies of NK receptor gene complexes will have to be interpreted in relation to their MHC ligands.

As more configurations of NK receptor genes are determined for different species, it will become possible to track the way groups of KIR and Ly49 loci have followed different species lineages. Were rodents the only species to lose KIR function? Are primates unique in losing Ly49? What are the intermediates on the way to “all KIR” and “all Ly49” models? And what selective advantages drove the specialization towards the KIR or Ly49 model in different species? 

## Abbreviations

CHIR: chicken Ig-like receptor
IgSF: immunoglobulin superfamily
ITAM: immunoreceptor tyrosine-based activation motif
ITIM: immunoreceptor tyrosine-based inhibitory motif
KIR: killer cell immunoglobulin-like receptor
LAIR: leukocyte-associated Ig-like receptor
LILR: leukocyte Ig-like receptor
LRC: leukocyte receptor complex
mCMV: murine cytomegalovirus
MHC: major histocompatibility complex
MIC: major histocompatibility complex class I chain-related protein
NK: natural killer
NKC: natural killer complex
SH2: Src homology 2
SHP[number]: Src homology 2 domain–containing protein tyrosine phosphatase [number]
